# Whole Genome Variant Dataset for Enriching Studies across 18 Different Cancers

**DOI:** 10.3390/onco2020009

**Published:** 2022-06-17

**Authors:** John Torcivia, Kawther Abdilleh, Fabian Seidl, Owais Shahzada, Rebecca Rodriguez, David Pot, Raja Mazumder

**Affiliations:** 1 The Department of Biochemistry & Molecular Medicine, The George Washington University Medical Center, Washington, DC 20037, USA; 2 Institute for Systems Biology-Cancer Gateway in the Cloud (ISB-CGC), General Dynamics Information Technology, Rockville, MD 20852, USA; 3 Independent Researcher, Glen Dale, MD 20679, USA

**Keywords:** TCGA, WGS, cancer variations, WGS analysis

## Abstract

Whole genome sequencing (WGS) has helped to revolutionize biology, but the computational challenge remains for extracting valuable inferences from this information. Here, we present the cancer-associated variants from the Cancer Genome Atlas (TCGA) WGS dataset. This set of data will allow cancer researchers to further expand their analysis beyond the exomic regions of the genome to the entire genome. A total of 1342 WGS alignments available from the consortium were processed with VarScan2 and deposited to the NCI Cancer Cloud. The sample set covers 18 different cancers and reveals 157,313,519 pooled (non-unique) cancer-associated single-nucleotide variations (SNVs) across all samples. There was an average of 117,223 SNVs per sample, with a range from 1111 to 775,470 and a standard deviation of 163,273. The dataset was incorporated into BigQuery, which allows for fast access and cross-mapping, which will allow researchers to enrich their current studies with a plethora of newly available genomic data.

## Simple Summary

Large quantities of whole genome sequences for paired tumor and normal tissue samples exist, but downstream processed data from these data are often not made available easily for further research. Our aim is to take 1342 of the whole genome sequence data generated by The Cancer Genome Atlas project and make them available as variant calls for downstream research, covering an average of 117,223 cancer-associated variants per sample pair. This set covers 18 different cancer types. We also show several different use cases for these data using the BigQuery tool.

## 1. Introduction

High-throughput sequencing technology has enabled a revolution in the field of genetics over the past few decades. In brief, this family of technologies allows the genome to be broken into small components and sequenced in a massively parallel way; an entire genome can be sequenced in a day instead of the decade it took to generate the first draft [1]. Scientists have been able to use these technologies to examine functional components of a whole genome, such as, most commonly, the exome, but also promoter and enhancer regions, and epigenetic markers [2]. Since the cost scales with the size of the genomic regions examined, scientists often focused on the much smaller, but more information-dense, exome region of the genome. Research has, however, shown that the non-coding region of the genome holds variants with explanatory power due to regulatory effects [3–6]. Now that costs have decreased dramatically [7] and laboratory consortiums have formed to pool resources, the use of whole genome sequencing has become more and more economically viable and more common in research projects.

The true cost of high-throughput sequencing includes both the raw material costs and the bioinformatics costs. Bioinformatics costs cover computational resources needed to store, analyze, and perform specialized tasks such as quality control and annotation on the data [8]. The costs associated with the analysis can now easily eclipse the raw material cost of a sequencing run due to the specialized training and computational resources required [9]. With these overhead requirements in mind, it is advantageous for researchers to share their processed data with each other to facilitate more rapid research in the field. Organizations have built infrastructures to facilitate the sharing and re-use of data. The National Cancer Institute (NCI), for example, has invested resources in its Cancer Research Data Commons (CRDC) to help drive research innovation [10–12].

In this spirit, we generated 1342 variant call datasets based on The Cancer Genome Atlas (TCGA) whole genome sequence (WGS) and alignment data. The dataset was constructed after analyzing the TCGA dataset for suitable whole genome sequencing experiments. The entries varied in their composition, so we focused on the unambiguous normal–tumor pairs, which left 2207 potential samples to analyze as shown in Table S1.

## 2. Materials and Methods

**Figure 1.**
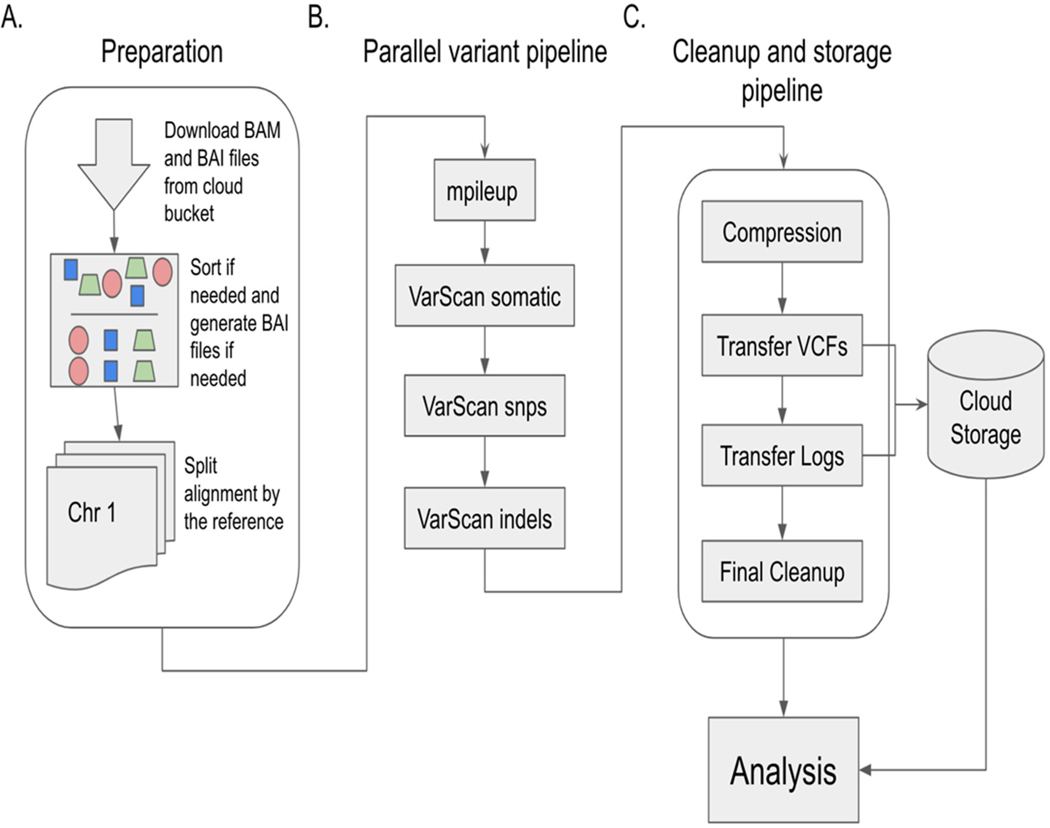
The pipeline used, encompassing the full lifecycle of generating the database. (**A**). The pipeline begins with a preprocessing step which includes downloading the alignment data (both the normal and the tumor alignments) as well as the index data for each through the Google Cloud storage bucket into the virtual machine to be processed. If needed, the alignment is sorted and an index file is generated (if one was not provided). The sorted alignment files are then split by chromosome in order to facilitate parallel processing. Since the data were often very large and the transfer speeds even between nodes were prohibitive, split alignments were kept to the local instance and parallelized by multithreading. Multiple nodes, however, were used to then parallelize across different tumor–normal paired samples. Each node had the human reference stored on disk and was not required to stream it for use. (**B**). The computational pipeline is run in parallel locally using the VarScan2 tool. Initially, SAMtools’ mpileup tool was used to convert the alignment files into an appropriate format for VarScan2. From here, VarScan2 was run to generate SNV and indel variations. These are each split into somatic, germline, and loss of heterozygosity, and further bifurcated by high confidence and low confidence. (**C**). The final step in the pipeline included housekeeping such as moving relevant generated files and logs to long-term storage and cleaning up the node in preparation for the next set of computations to be run. This step happens once all chromosomes are handled in the VarScan2 pipeline used.

TCGA WGS data are available in alignment form for cancer-related studies but are not always further processed into variant calls. We used the pipeline shown in Figure 1 to generate variant data covering 1342 normal–tumor sample pairs as described in Table S2.

**Figure 2.**
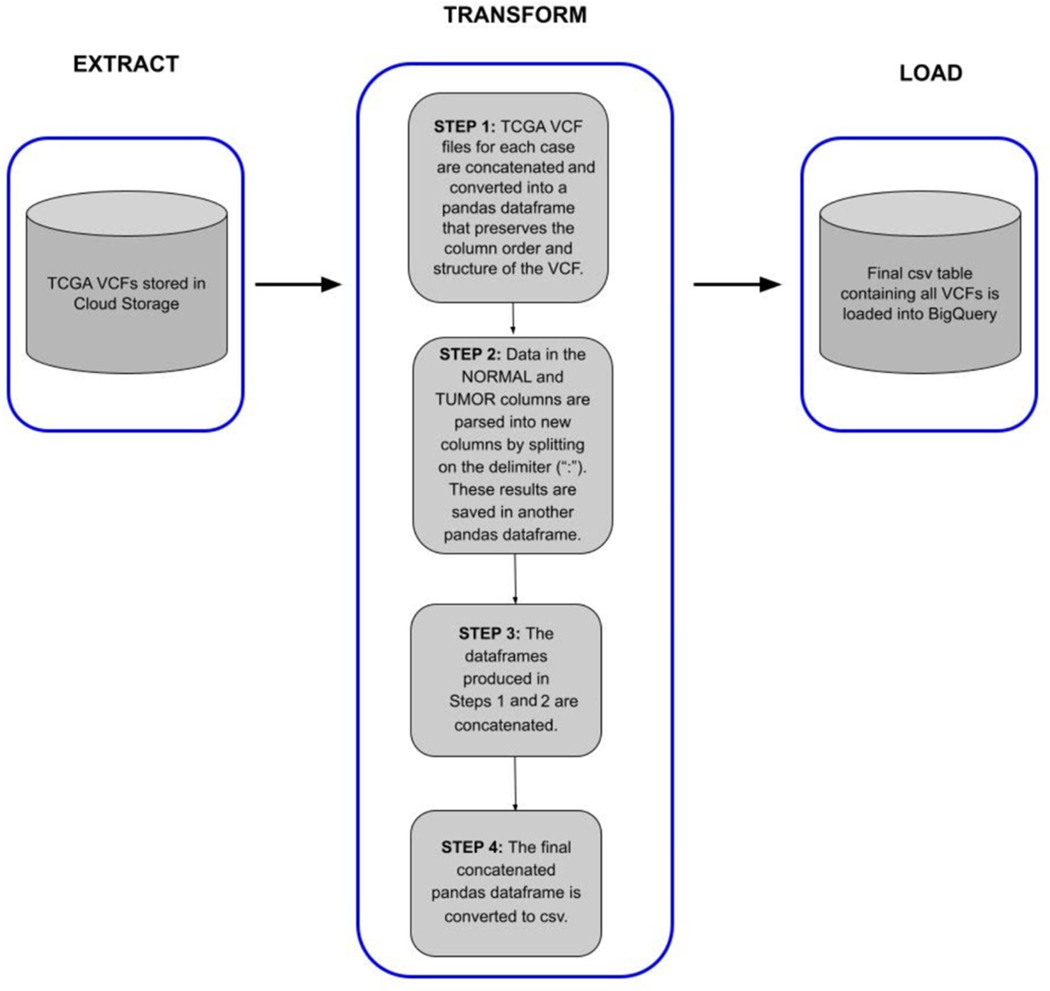
The ISB-CGC VCF2BQ ETL pipeline produces Google BigQuery tables that serve as central repositories for VCF files. In this analysis, we used the ETL pipeline to transform TCGA VCFs generated from the pipeline described in Figure 1. Variant data in one central BigQuery table afford the ability to query and interrogate the data without the need to download. In addition, the ETL process maintains the column composition of the VCF file format.

After generation of the processed data, we performed an ingestion pipeline as shown in Figure 2, to convert the data from VCF into a BigQuery table, to facilitate fast and flexible searching and splicing.

### 2.1. Data Generation

The NCI CRDC cloud-enabled data ecosystem, through three different cloud resources, provides access to large data in a colocalized way to facilitate faster analysis. ISB-CGC was the chosen cloud resource for this analysis since it provides direct access to the data outside of a set framework. Due to the large amount of data being analyzed, it was necessary to use a custom framework to both exploit the highly parallelizable nature of the work and to manage computational resources appropriately. Data access was facilitated through virtual machine instances on Google Cloud through the ISB-CGC cancer cloud.

#### 2.1.1. Targeted Data

The goal of the original study [13] was to look at the non-coding region of the genome in normal–tumor paired samples in a functionally agnostic way. The approach required variant calling on the entire genome and is expanded here to include a larger sample of the data within TCGA.

All possible samples were examined for inclusion in the expanded dataset. Samples that were not typical normal–tumor pairs were removed from the data generation, leaving behind a set of 2207 unambiguous normal–tumor pairs from the TCGA data (Table S1). For various reasons, not all 2207 were successfully processed. Largely, this was due to extremely large alignment files that would have required more computational resources than were available. A total of 1342 samples were successfully called without computational error and passed validation, and are shown in Table S2 with SNV counts.

#### 2.1.2. Variant Calling Methodology

The alignment procedure from raw sequence data to BAM files was conducted by the TCGA network. The methods for this process are outlined online. Variant calling on normal–tumor pairs was performed with VarScan2 software, as outlined in the pipeline in Figure 1.

### 2.2. Custom Submission Framework

Utilizing Google Cloud Engine, we constructed a management system that retrieved appropriate data, ran the VarScan2 pipeline in parallel, and then captured the relevant data. SLURM [14] was chosen as the scheduling software for this task and both an analytics server and database interface were written in Python (see Code Availability below). Multiple nodes were set up on Google Compute Engine to support this orchestration. These consisted of: 1 compute engine datastore, 1 SLURM master node, 1 analytics node, and 50 compute nodes. Protected TCGA data were provided through ISB-CGC after authentication and authorization, and were transferred onto compute nodes as needed.

A custom Python interface was written for the SLURM master node in order to easily facilitate submission of jobs based on TCGA ID codes (see Code Availability below). An arbitrary number of jobs could be submitted and queued through SLURM with this setup. The interface with the SLURM master accepted a comma-separated value (CSV) file with information related to the TCGA samples to be queued. The generated job set included (1) a single job per chromosome, (2) one for initial downloading and splitting of the sample, and (3) one for cleanup and storage of the results. Each of the computational nodes was set up with necessary tooling including SAMtools [15] and VarScan2 [16–18].These were installed as per their installation instructions.

#### 2.2.1. Download and Split

The first job run was the download and split job performed by a custom bash shell script. This would download all appropriate files for the job through the Google-provided gsutils tool, copy custom scripts to the compute node, and then split the alignment data by chromosome. Progress was reported to the analytics server through a custom script. Split alignment files were then staged on the compute node for both the normal and tumor samples.

#### 2.2.2. Variant Calling

The variant calling computations were performed next through the VarScan2 pipeline with individual chromosome references. After the first job was completed, which fetched the data, these chromosome-specific jobs were run in parallel. Progress was reported during each step to the analytics server. In short, the supporting scripts performed the following:

1. SAMtools mpileup
  samtools mpileup -f ${REFERENCE} -q 1 -B ${SORTED_NORMAL} ${SORTED_TUMOR}1> ${MPILEUP_OUTPUT}
  where ${REFERENCE} is the appropriate chromosome reference file, ${SORTED_NORMAL} is the sorted normal tissue alignment (BAM) file, ${SORTED_TUMOR} is the sorted tumor tissue alignment (BAM) file, and ${MPILEUP_OUTPUT} is the output file for this intermediate pileup file.
2. Base Somatic Mutation Calling through VarScan2
  java -jar VarScan.jar somatic ${MPILEUP_OUTPUT} ${BASE_OUTPUT} –mpileup 1 –min-coverage 8 –min-coverage-normal 8 –min-coverage-tumor 6 –min-var-freq 0.10–min-freq-for-hom 0.75 –normal-purity 1.0 –tumor-purity 1.00 –p-value 0.99–somatic-p-value −.05 –strand-filter 0 –output-vcf
  where ${MPILEUP_OUTPUT} is the output file from the SAMtools mpileup step, and ${BASE_ OUTPUT} is an arbitrary output file name.
3. Somatic SNV calling
  java –jar VarScan.jar processSomatic ${OUTPUT}.snv.vcf –min-tumor-freq 0.10 –max-normal-freq 0.05 –p-value 0.07
  where ${OUTPUT} is the output file name from the base somatic mutation calling.
4. Somatic indels calling
  java –jar VarScan.jar processSomatic ${OUTPUT}.indel.vcf –min-tumor-freq 0.10 –max-normal-freq 0.05 –p-value 0.07
  where ${OUTPUT} is the output file name from the base somatic mutation calling.

#### 2.2.3. Cleanup and Storage

Once all variant calling jobs were complete, a cleanup and storage script was run. This program performed various housekeeping functions such as compressing the output files, moving them to the storage bucket, copying error output and other logging to storage, and posting progress to the analytics server. This job then removed the staging directory on the compute node, freeing it up to begin the next sample in its queue.

### 2.3. Data Ingestion into BigQuery

The ISB-CGC VCF to BigQuery (VCF2BQ) pipeline provides a method to extract, transform, and load (ETL) VCF files stored in Google Cloud Storage buckets to Google BigQuery tables. This script was provided by ISB-CGC in order to automate some of the effort required to upload data to BigQuery within ISB-CGC. The primary purpose of the script is to preserve the integrity of the VCF file format most researchers are familiar with while splitting some of the columns to simplify queries and leverage the power of BigQuery data analytics. The script, implemented in Python, uses various Python data structures and libraries to transform and load VCF files into BigQuery tables. The script is optimized to handle VCF files derived from tumor–normal variant calling algorithms, but can also be used for more general VCFs as well.

#### Implementation

Each VCF file is converted into a pandas data frame that contains columns that preserve the variant record information as well as columns of metadata about the files, including TCGA Genomic Data Commons (GDC) identifiers (case_barcode, sample_barcode, case_gdc_id, file_gdc_id) along with information about variant caller (e.g., SomaticSniper, Muse, etc.). These columns serve as unique identifiers that can be used by researchers to locate more information about the files in the GDC data repository to join with other data types in BigQuery (see example notebook). The script can execute on single files or on bulk VCFs at once. All generated pandas data frames are concatenated into a large dataframe, which is loaded into BigQuery. The final VCF BigQuery table can be used for downstream analysis using either Google BigQuery’s standard Structured Query Language (SQL) or can be further interrogated in R or Jupyter Notebooks. The script is maintained in the ISB-CGC ETL GitHub repository.

The VCF2BQ Script was used to extract, transform, and load controlled-access TCGA WGS VCF generated using the Varscan2 pipeline. dbGAP authorization for TCGA data is required to access the VCF files.

The script is optimized to run on the Google Cloud Platform, leveraging Google’s Compute Engine, Google BigQuery, and Google Cloud Storage Buckets. Optimally, the script is executed on Google Compute Engine VM instance because of the fast transfer speeds between virtual machines and cloud storage.

## 3. Results

In the original analysis [13], five different TCGA cancer types were covered over 154 samples. This paper aimed to extend this to 1386 samples covering 18 different cancer types in the TCGA dataset (see Table 1 for cancer code definitions used by TCGA and within this paper). Of this number, 1342 passed validation (see Validation section, Supplementary Table S2 for successful samples, and Supplementary Table S3 for failed samples), leaving 44 entries that failed quality control.

**Figure 3.**
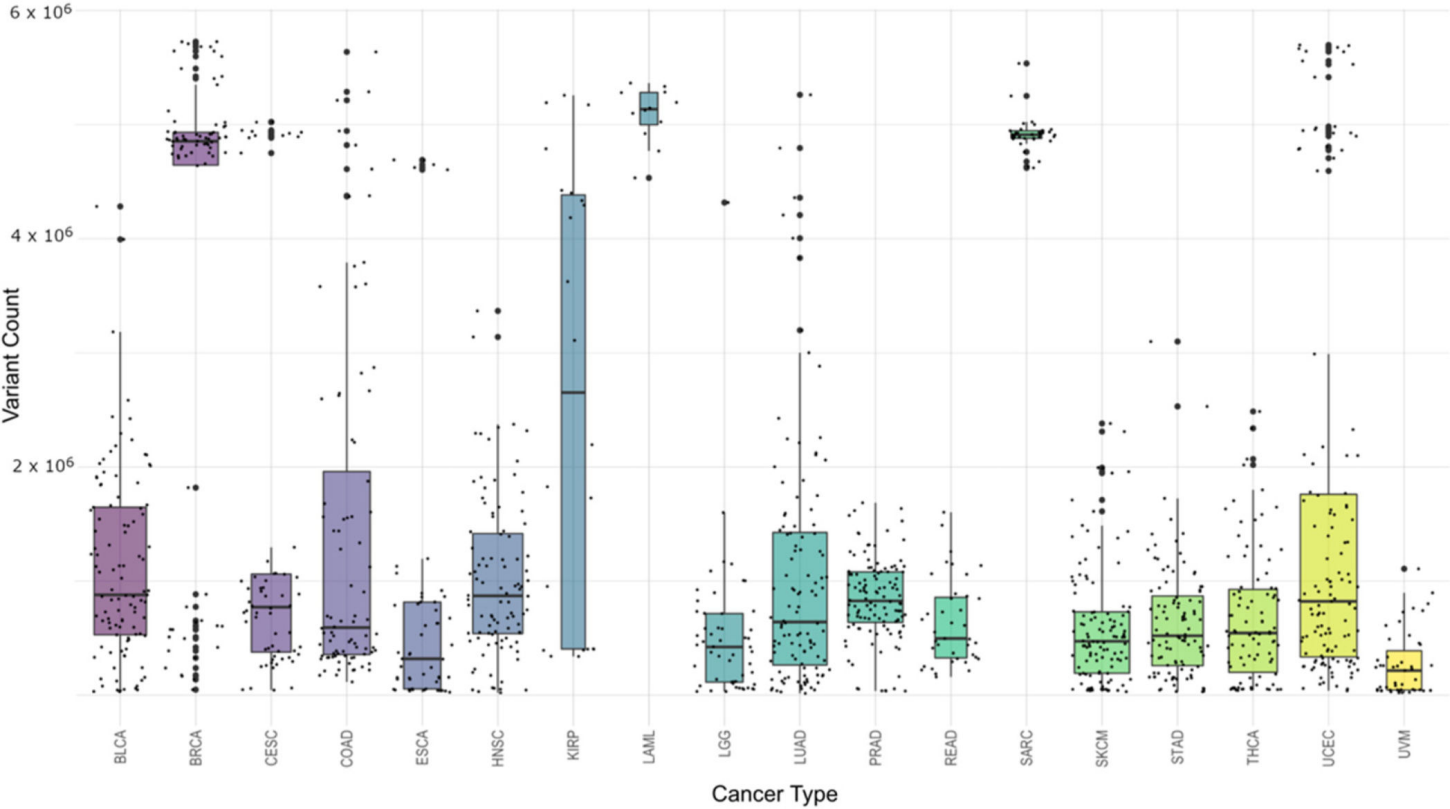
Distribution of variants within each cancer type. Various cancer types, in this dataset, exhibit different ranges of normal–tumor pair variations as shown here. For each cancer type represented by the TCGA cancer code (see Table 1 for definitions), the number of cancer-associated variants is plotted with the count being defined on the Y axis. Within each column representing a single cancer, the counts are plotted with an offset for readability purposes. Further, box and whisker plots are drawn showing the quartiles and the means across all of the samples for a particular cancer type.

Final data generation revealed 157,313,519 pooled (non-unique) cancer-associated single-nucleotide variations (SNVs) across all samples. This was an average of 117,223 SNVs per sample with a range from 1111 to 775,470 and a standard deviation of 163,273, illustrating the variation in sample preparation and experimental design decisions made by the participating laboratories. Figure 3 shows the distribution of variant counts within each cancer type. One thing that is important to note here is that the results from this cohort are unique to this study and should not be viewed as summaries for that cancer type in general. Due to the differences in read depth between different cancer types as well as different research goals set before sequencing, the numbers represent only a snapshot of this particular set and not the cancers as a whole.

**Figure 4.**
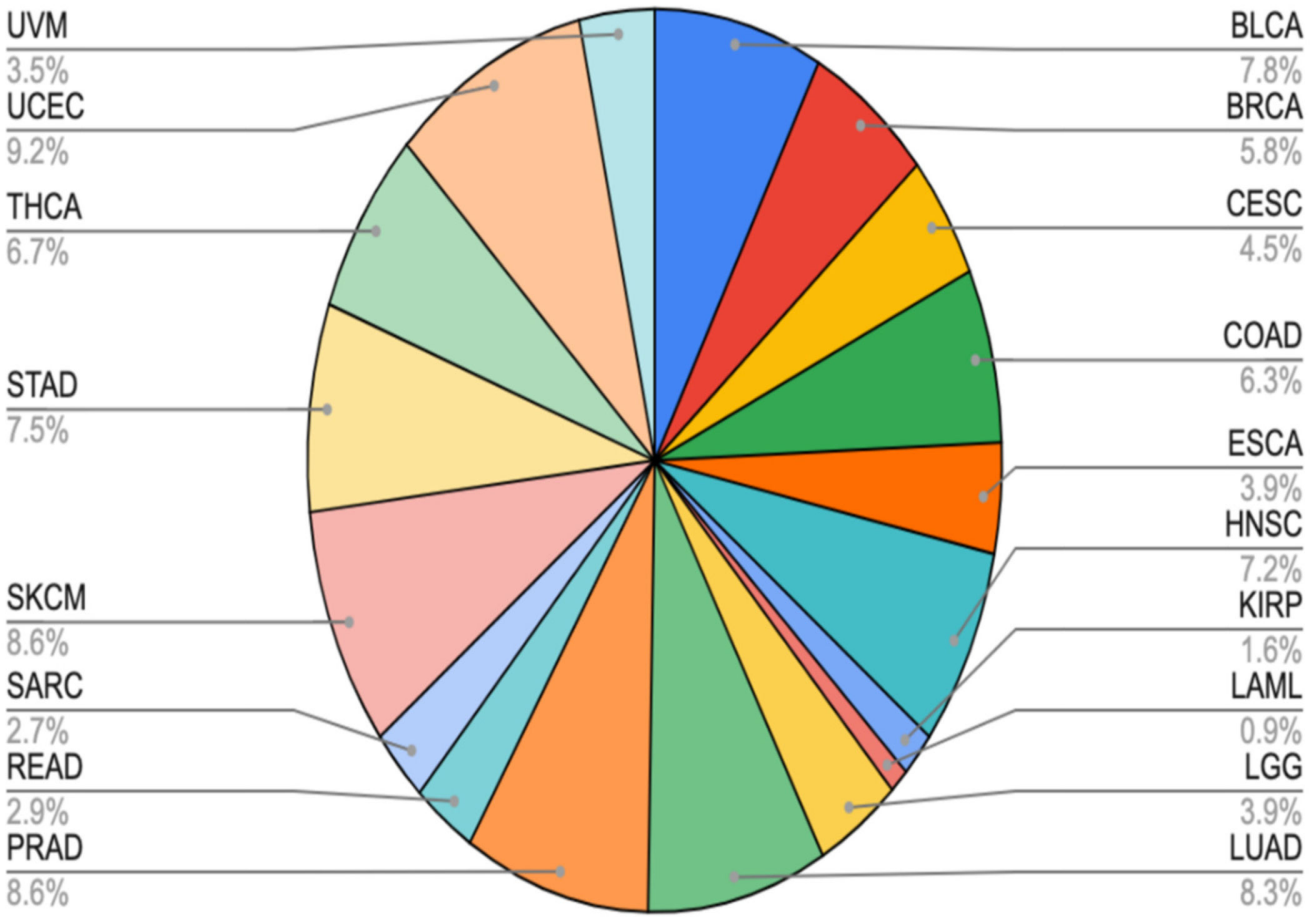
Makeup of the dataset. The distribution of the normal–tumor pair samples across the successfully called samples. This shows a relatively even distribution across all of the captured cancer types. Note that the total for the pie chart is 99.9% for the numbers shown due to rounding them to a single decimal place for readability.

The makeup of the dataset ranges from Acute Myeloid Leukemia (LAML) at 0.89% of the samples to Uterine Corpus Endometrial Carcinoma (UCEC) at 9.17%. While this range is large, the average of each of these 18 cancer types was 5.56%, the median value was 6.04%, and the standard deviation for the ratios was 2.54. The sample distribution by cancer type is relatively balanced, as seen in Figure 4, which will lend itself to many different types of analysis.

**Figure 5.**
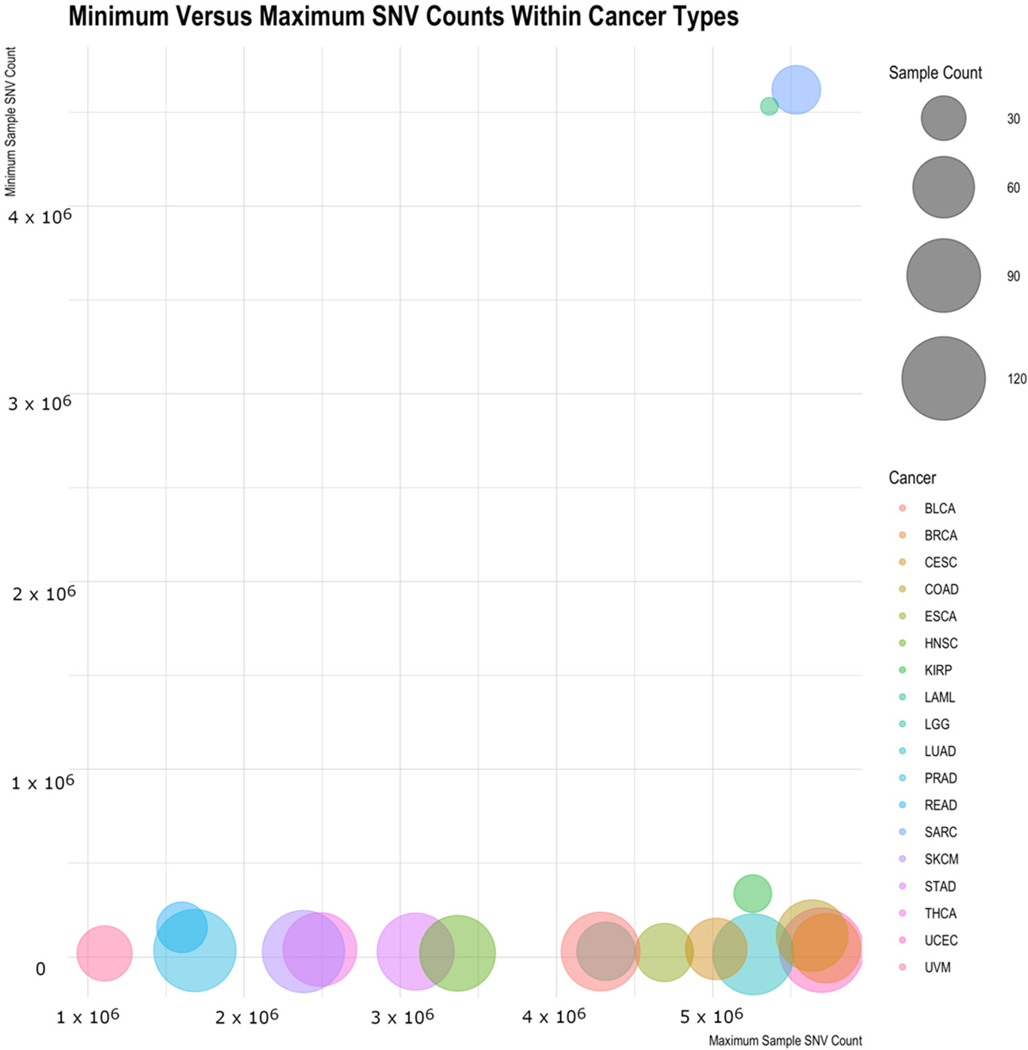
Minimum sample variant count for each cancer type versus the maximum variant count, with the average variant count being the size of the bubble. SARC and LAML appear to be outliers when examining this ratio.

When looking at the distribution of number of variants across cancers, a slightly different picture emerges, as there are a few outliers. Table 2 shows for each cancer type the mean and median number of variants per sample, the standard deviation within the cancer type, the minimum and maximum variant counts, and the sample counts. Both Acute Myeloid Leukemia (LAML) and Sarcoma (SARC) are interesting in that they have high minimum values (the sample of that cancer type with the fewest variations) and have low standard deviations. While the standard deviation scores could be in large part due to the limited sample size, the minimum cancer-associated variations for these samples are intriguing and could suggest something biologically distinct for them. Figure 5 illustrates this by showing the minimum sample variant count for each cancer type versus the maximum variant count, with the average variant count being the size of the bubble. LAML and SARC look to be outliers to the rest of the cancers. The general trend shows the minimum counts to be independent of the maximum count and the average SNV count generally not related to the maximum (or the minimum) count.

**Figure 6.**
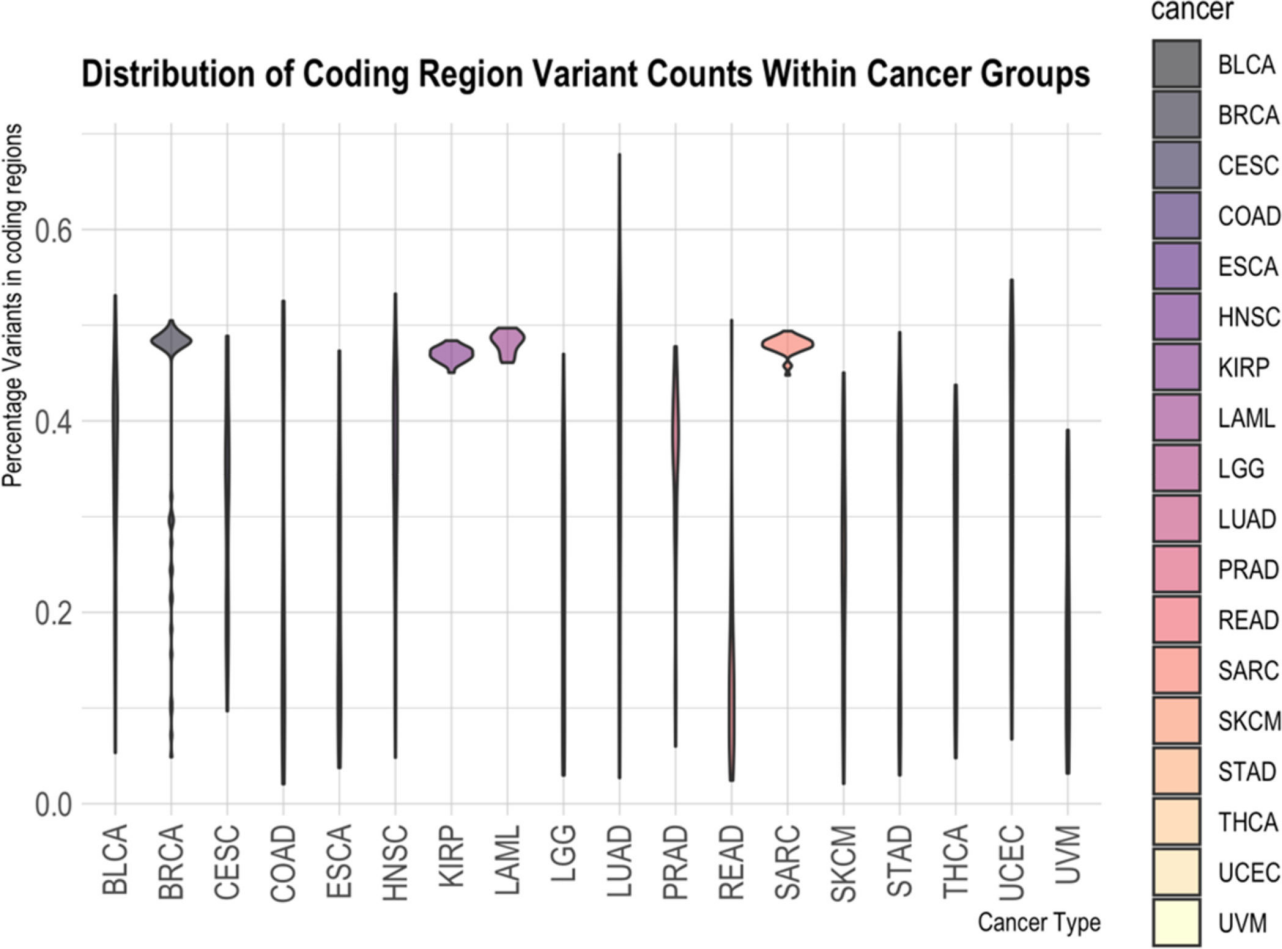
Violin plot of the percentage of variants found within the coding region within each sample. Grouped by cancer type on the x-axis, the ratio of variations in the coding region is shown on the y-axis. For most of the cancer types examined, the samples had a wide variety of coding vs. non-coding ratios (SNVs found in CDS regions/total SNVs excluding mitochondria). SARC, LAML, and KIRP show an interesting pattern where their ratios are highly contained to a small region.

Looking next at the non-coding region of the genome, we see in Figure 6 that most cancer types analyzed in this study have widely different amounts of variation found in the non-coding region versus the coding. The percentages of coding SNVs were calculated by taking the raw counts of variants found in CDS regions [19–21] and dividing by the number of variants found in total (excluding mitochondrial variants since consensus mitochondrial coding regions are not included in the dataset used). The wide variety of ratios found through the samples in most cancers is in stark contrast to SARC, LAML, and KIRP. These three cancers exhibit, within this study, the property that all of their samples have nearly the same ratio of coding to non-coding and also have nearly the same number of non-coding and coding variations.

The SNV calls provide valuable information on cancer-associated changes in the genome outside of the original research driven by them [13]. In order to facilitate contributing this information back to the scientific community, we have deposited the processed output with the NCI Cancer Data Service (CDS). The submission is registered with the Database of Genotypes and Phenotypes (dbGaP) and is accessible in CDS to authorized researchers through their cloud service (https://datacommons.cancer.gov/repository/cancer-data-service, accessed on 1 June 2022). Searchable tables for metadata related to the samples are available via the Institute for Systems Biology (ISB)’s Cancer Gateway in the Cloud (ISB-CGC) (https://isb-cgc.org, accessed on 1 June 2022), one of NCI’s Cloud Resources (https://datacommons.cancer.gov/analytical-resource/isb-cancer-gateway-cloud, accessed on 1 June 2022) [22].

### 3.1. Data Records

The pipeline as described in the methods section was run on normal–tumor paired samples from TCGA. There were several records generated through this pipeline with high-confidence somatic single-nucleotide variations (SNV) calls being deposited to the CDS cloud repository.

These high-confidence somatic SNVs were generated for each of the processed samples as a standard VCF file and ingested into BigQuery as a single table across all samples. Raw VCF files can be regenerated from a BigQuery table if needed after ingestion (see Usage Case 3 in this paper).

The SNV dataset shows individual positions in the genome where a nucleotide in the cancer tissue differs from the reference genome (hg19) but not the paired tumor tissue. Both the normal and tumor samples were also required to have adequate coverage. In the current pipeline, this translates to a read depth of 8 for normal tissue reads and 6 for tumor tissue reads at the location of the SNV call. VarScan2 calls SNVs by using a heuristic method and performing a statistical test which considers the number of aligned reads for each allele [20–22].

### 3.2. Technical Validation

We pursued several strategies to assess the quality of the data and to correct for errors that are common in highly parallelized pipelines. After the variant calling pipeline was finished, the output data were a compressed archive of different sets of information including SNVs, indels, germline mutations, error logs, etc. If a computation failed, the size of the output archive would be approximately 10 kb. This allowed for quick screening for failed computations that needed to be rerun or investigated further. This was a semi-frequent occurrence for several reasons. The data were controlled and sometimes required significantly more time to transfer than our access window, occasionally causing transfer issues.

Additionally, as each step of the computation was run, we were reporting progress and command output values to a database being run in the cloud cluster. This database accepted reporting for a wide variety of values including error codes of interest and computational metrics for future regression analysis. Examining the database was useful for diagnosing computational problems as they occurred and allowed for on-the-spot corrections. These corrections were often re-running the sample due to I/O issues or file corruption.

Variations were mapped to 10,000 base windows to bucket the variations for visualization purposes. These windows were plotted in a Manhattan plot-type graph with region number and chromosome on the x-axis, while the y-axis was the number of variations found in the region. Computations that failed to produce expected results across the genome would have entire chromosomes missing, which indicated a computational problem. Samples which failed this test were re-run and all were successfully recovered.

To shield against outliers, a strategy was taken to limit the right-tailed variations to being within a standard deviation of the average variant counts across the samples. Since an alignment cannot produce fewer than zero variations, the data are right-skewed but the validation would allow the left side to be arbitrarily low and only check against the right side. This is because we would not necessarily expect an arbitrary low count of variations to be an outlier since it is possible that only a handful of variations could lead to cancer. On the other hand, it is difficult to imagine that millions of variations would be required for oncogenesis and more likely this related to mis-matched samples (or some other error). In any case, with the average total variation count (not restricted to high-confidence somatic to capture a broader section of outliers) being 1,651,974 and the standard deviation being 4,090,131, no left-side values would fail the test even if they were not excluded. All variant counts that then fell above 5,742,105 variations were discarded as outliers using this methodology and are reported in Table S3. This was a total of 44 out of the 1386 finished computations.

An additional strategy used for quality control was that all downloads were checked against their md5sum hashes (mathematical representations of data that differ even if a single bit is changed in the data) to verify there was not a download-related corruption issue. There were several samples that were only partially transferred during the download, and a simple retransfer was able to correct this.

A final validation was performed after the BigQuery tables were ingested. The tables were validated to have the expected number of samples and verify that variant counts matched what was seen in the raw VCF files.

## 4. Discussion

Here, we present a dataset which covers 1342 WGS cancer-associated SNV calls across 18 different cancer types as defined by TCGA. These data cover an assortment of variants within the whole genome and offer an opportunity for researchers to deeply dive into differences between normal and cancerous tissue within the same patient.

The pipeline itself, as illustrated in Figure 1, can be used by researchers as a blueprint to run their own analysis on normal–tumor paired data either from the TCGA or other studies. The software provided in the associated GitHub repositories can be modified to support non-TCGA content and accelerate the computational component to many research questions by lowering the barrier to performing these computations.

Currently available datasets through TCGA provide only whole exome sequencing (WXS)-level data. While there is a high number of exome sequences for normal–tumor paired samples as a result of the TCGA project, whole genome sequences have remained in raw format. This publication presents the cancer-associated SNVs for many of the WGS samples published by TCGA. With this dataset in hand, researchers can supplement their current research with these enriched data without bearing the high computational and financial cost of determining the variants themselves.

From this study, SARC and LAML data within the TCGA project are interesting outliers. All the samples within these cancers have an unexpectedly high number of variants compared with all other cancers in the set. The fact that they appear to be outliers requires a closer look at the specifics of the data collection and sequencing for these cancers before using them in further studies as they may be inappropriate datasets for some contexts. Other cancer types have many samples with strikingly low numbers of variants. These samples tend to be ones collected early in progression. This matches expectations as younger cancers would have less time to accumulate mutations. An upper level or ceiling on variations would not be expected since these samples were taken at various stages of cancer growth and therefore could have had significant time to continue to mutate. This finding provides confidence on using those specific cancer-associated variations.

These two cancer types, along with KIRP, also show some interesting differences compared to the other cancer groups with reference to non-coding vs. coding variation counts. Within this study, the samples in these cancer types have very close percentages of coding variants out of the entire variant set. They also have nearly the same ratio of non-coding to coding variations. This implies that the methodology used for these cancers in the TCGA project may have had some bias in it. Alternatively, it could imply that there is something particular about these types of cancers which weighs variations in this way. Whether or not non-coding variations have a special meaning for these cancers is an interesting research topic.

The ability to easily use this dataset was one of the priorities of the authors during this study. Several exemplary ways to use the variant datasets are examined below.

### 4.1. Use Case 1: Determination of New Entries to a Cancer Database

Cross-mapping between annotation and variant databases is a common use case to increase the value of the variant database. In addition to annotation and variant crossmappings, a similar approach is used to enrich a variant database with additional entries. The first use case was to take an existing cancer variant database and determine, quickly, how many variants are within the new dataset that are not represented in the existing dataset. We used the BioMuta database [23], which primarily focuses on exome region variants. As a result of this, we expected that there would be many new variants found in the high-confidence somatic dataset published in this paper.

We first ingested BioMuta into BigQuery as a custom dataset, following instructions for BigQuery. From the BigQuery web interface, we constructed a SQL command to map the high-confidence somatic variations to the BioMuta database to determine how many variants found from this project are not yet represented in the BioMuta database. While constructing the SQL command can seem daunting, it uses a relatively easy-to-learn structured query language (SQL) with which many scientists are already familiar.

An example SQL command is provided here, although there would need to be slight modifications depending on the details of the BioMuta ingestion.


SELECT v4_0.*
     FROM biomuta.v4_0
          WHERE NOT EXISTS(SELECT *
                FROM ‘isb-cgc-04–0026.fs_scratch.tcga_variants’ WHERE CHROM = CONCAT(“chr”, CAST(v4_0.chr_id as string)) AND POS = v4_0.chr_pos);


This command retrieves from the high-confidence somatic variant BigQuery table all of the variants that exist there but do not exist in the BioMuta version 4 dataset. The output from this command will return a table with all entries in the somatic high-confidence variant table that are not found in BioMuta. The count of rows in this table is the number of variants that are not represented in the BioMuta dataset. We found that there were 7,630,735 (non-unique) variants which were not found in the BioMuta dataset. The duration of the BigQuery search creating this mapping was 18.3 s.

### 4.2. Use Case 2: Generation of Summary Statistics of the Dataset

Summary statistics of a dataset can easily be generated by using either the BigQuery API and generating via Python (or some other language) or generated through the BigQuery language itself. A simple example is to generate the counts of high-confidence somatic variations for each cancer type.


SELECT COUNT(CHROM), project_short_name
         FROM ‘isb-cgc-04–0026.fs_scratch.tcga_variants’
         GROUP BY (project_short_name);


This SQL command will generate a table of each of the cancer types as well as the variation counts found in the somatic high-confidence table. It performs this task by reading the project name and counting the number of hits for each, and then presenting the results as the output from the command. The project counts from this use case are included in Table 3.

### 4.3. Use Case 3: Regenerate a VCF File from the BigQuery Tables

While the data as published in BigQuery tables are useful for cross-table investigation, it is often required to have a VCF-formatted file for a specific pipeline where the software is expecting that format. A standard formatted VCF file can be directly generated from the BigQuery tables, as needed.

Unlike the other use cases, this case requires the output from the BigQuery table as the input and then processes it into a standard VCF-format file. The table output can be retrieved either through the BigQuery interface by running a general fetch command focused on the sample of interest (shown below) and saving the output table in comma-separated value (CSV) format through the BigQuery tools, or through the BigQuery API in Python or another language (not shown).

SELECT * FROM ‘isb-cgc-04–0026.TCGA_WGS_HG19_VCF.somatic_hc_variants’ WHERE project_short_name) = ‘TCGA-44–2656’

Once the input data have been generated, a simple script can be used to convert these data into a VCF file (see Code Availability below) by generating the VCF header text and then looping through each of the entries and outputting into the appropriate VCF formatting. This script accepts a TCGA ID as a required parameter and can also be given a specific chromosome and a limit to the number of SNVs returned, if desired. Specific instructions on running the script are provided in the repository.

### 4.4. Study Limitations

While this study presents a large, cancer-associated SNV dataset there are several limitations which should be noted. First is sample size. Even 1342 whole genome cancer variant sets are likely to be insufficient to untangle cancer comprehensively. Cancer is an umbrella term capturing diseases of many different tissues; further, even within these categories, each individual cancer can be caused by different mechanisms of cellular dysregulation. Therefore, it is unlikely that even a dataset of this size will have enough explanatory power to answer all biological questions related to even a single type of cancer. The hope is that this dataset, along with many others that are produced, can help drive understanding of this disease when supplementing ongoing research.

The dataset is not a comprehensive processing of all of the TCGA sequencing data. There are around 2200 whole genome normal–cancer pairs within the consortium’s data, meaning this dataset includes 60% of the TCGA cancer-associated variants. For several different reasons including computational, logistical, and a conservative approach to the data that were in question, the entire set was outside of the scope of this experiment. This represents both a limitation of this dataset as well as an opportunity for additional data available to supplement more specific research questions.

Additionally, TCGA data represent a single coherent study and may not represent all cancer data. The consortium study had various standards for data collection and analysis which are incredibly useful for comparing data between the different laboratories, but also run the risk of biasing the entire dataset in some way. Care will need to be taken when combining this dataset with others to make sure they are compatible.

Any insights gleaned from the dataset would need to be validated against real-world samples. A purely data-driven approach can only point us in the right direction for research but cannot currently replace validation-level research that would be required in a clinical setting.

The TCGA project utilized short-read-focused, next-generation sequencing which, while revolutionary, does not offer a comprehensive genetic profile of the genome. Longer read technologies help in examining copy number variation, different techniques are used for 3D mapping of the genome, non-coding rearrangement, and other block rearrangement of the genome, and many other techniques are under development to see beyond the nucleotide-level sequence. All of these techniques, and more, would be useful in a full examination of the cancer genome. This study reports on the short-read-focused results and is therefore limited to the information captured by these techniques.

Finally, despite the high-confidence estimates used in the study, there are likely to remain false positive variants in this set. It is possible that many of these have no or very low impact, which may be difficult to deduce without a large quantity of cancer genomic data, far beyond this study.

Even with these limitations, the dataset of 1342 whole genome, cancer-associated, high-confidence SNV calls provides an exciting opportunity for researchers to supplement their current and future studies.

## Supplementary Material

Supplementary

## Figures and Tables

**Table 1.** TCGA uses codes for various cancer types as part of its ontology. The TCGA codes used in this paper as well as the standardized cancer names are included in this table for reference.

TCGA Code	Cancer Name
BLCA	Bladder urothelial carcinoma
BRCA	Breast invasive carcinoma
CESC	Cervical squamous cell carcinoma and endocervical adenocarcinoma
COAD	Colon adenocarcinoma
ESCA	Esophageal carcinoma
HNSC	Head and neck squamous cell carcinoma
KIRP	Kidney renal papillary call carcinoma
LAML	Acute myeloid leukemia
LGG	Brain lower-grade glioma
LUAD	Lung adenocarcinoma
PRAD	Prostate adenocarcinoma
READ	Rectum adenocarcinoma
SARC	Sarcoma
SKCM	Skin cutaneous melanoma
STAD	Stomach adenocarcinoma
THCA	Thyroid carcinoma
UCEC	Uterine corpus endometrial carcinoma
UVM	

**Table 2.** Summary statistics of the dataset. This table shows a number of summary statistics across each of the cancer types based on the number of variations reported in the high-confidence somatic SNV pipeline.

Cancer	Mean	Median	Standard Deviation	Min	Max	Sample Count
BLCA	1,115,860	878,361	828,726	30,469	4,282,066	105
BRCA	3,914,925	4,853,067	1,962,690	47,614	5,725,487	78
CESC	1,409,171	771,086	1,705,045	44,244	5,022,570	60
COAD	1,402,421	591,772	1,504,539	115,715	5,635,942	84
ESCA	789,184	318,753	1,302,847	25,368	4,689,557	53
HNSC	1,009,823	870,112	695,101	21,215	3,366,516	96
KIRP	2,643,152	2,651,145	1,952,154	339,066	5,255,891	22
LAML	5,088,546	5,134,114	246,890	4,532,075	5,362,914	12
LGG	517,464	421,754	645,682	32,004	4,316,099	53
LUAD	1,037,623	640,943	1,086,595	15,672	5,259,861	112
PRAD	836,010	825,482	361,895	35,700	1,685,032	116
READ	634,840	496,493	378,566	159,711	1,602,407	39
SARC	4,916,603	4,910,429	151,412	4,619,782	5,534,903	36
SKCM	595,721	471,930	526,485	30,378	2,380,354	116
STAD	636,949	520,248	507,724	30,274	3,098,160	100
THCA	672,999	544,240	578,580	41,538	2,485,252	90
UCEC	1,579,253	821,528	1,778,498	37,717	5,696,505	123
UVM	267,324	214,871	279,506	20,541	1,106,908	47

**Table 3.** The results from Use Case 2. This table shows the cancer type with the count of variations found within the table across all samples pooled together.

Project Short Name	Variant Count
TCGA-UCEC	17,803,998
TCGA-HNSC	7,492,300
TCGA-READ	507,962
TCGA-STAD	5,280,745
TCGA-PRAD	8,266,914
TCGA-KIRP	9,305,100
TCGA-THCA	4,650,240
TCGA-BRCA	33,526,277
TCGA-LGG	2,410,086
TCGA-LUAD	8,860,456
TCGA-BLCA	8,187,198
TCGA-COAD	8,715,859
TCGA-CESC	8,075,254
TCGA-ESCA	4,026,382
TCGA-UVM	1,081,309
TCGA-SARC	18,088,980
TCGA-SKCM	5,550,475
TCGA-LAML	5,483,984

## Data Availability

Access to the data is controlled as specified by The Cancer Genome Atlas project, but is otherwise available for authorized researchers through the Database of Genotypes and Phenotypes (dbGaP) and the NCI Cancer Data Service (CDS).

## Footnotes

Code Availability Source code and details for the TCGA analysis pipeline can be found in the repository located at https://github.com/syntheticgio/tcga_varscan_pipeline (accessed on 20 September 2020). Example code used in this paper for summary statistics and use cases can be found in the repository located at https://github.com/syntheticgio/wgs_to_vcf (accessed on 1 June 2022). Finally, the ISB-CGC resources for BigQuery ingestion are hosted at https://github.com/isb-cgc/NextGenETL (accessed on 1 June 2022). There are no access restrictions to the code provided, but access to the data is controlled as specified by The Cancer Genome Atlas project.
