# Supplementary material for: Whole Genome Variant Dataset for Enriching Studies across 18 Different Cancers
Source: Onco (Basel). Author manuscript; Available in PMC 2023 Oct 13. (PMC10571071; doi:10.3390/onco2020009)
Supplement: Supplementary [file NIHMS1921915-supplement-Supplementary.zip › Supplementary Table Legends.pdf]

Supplementary Table S1. List of all normal-tumor paired matches considered for variant calling from the TCGA dataset. Only patients which were deposited with unambiguously normal-tumor pairs were included, resulting in a potential of 2207 matched samples. Other samples (which included ones which lacked a normal or tumor sample, ones which had multiple normal/tumor samples, etc. were excluded since they would have warranted a different approach). Information in the table includes the cancer type, the TCGA IDs of each of the matched samples along with their locations in a Google Storage bucket (controlled access).

Supplementary Table S2. The final list of accepted VCF files along with the number of Somatic SNVs in each sample pair. Total SNVs counts for accepted VCF files provided as a second sheet.

Supplementary Table S3. The final list of rejected VCF files, along with the number of SNVs (if calculated) in each sample pair. Ones which have ”NA” represent samples where the counts were above 1 billion and not computed.
